# Characterization of pure cultures isolated from sulfamethoxazole-acclimated activated sludge with respect to taxonomic identification and sulfamethoxazole biodegradation potential

**DOI:** 10.1186/1471-2180-13-276

**Published:** 2013-12-01

**Authors:** Bastian Herzog, Hilde Lemmer, Harald Horn, Elisabeth Müller

**Affiliations:** 1 Chair of Urban Water Systems Engineering, Technische Universität München, Am Coulombwall, D-85748 Garching, Germany; 2 Bavarian Environment Agency, Bürgermeister-Ulrich-Str. 160, D-86179 Augsburg, Germany; 3 Karlsruhe Institute of Technology, Engler-Bunte-Institut, Bereich Wasserchemie und Wassertechnologie, D-76131 Karlsruhe, Germany

**Keywords:** Batch setups, Wastewater treatment plants, Xenobiotics, UV-absorbance, Pharmaceuticals, Phylogenetic analyses, Sulfamethoxazole

## Abstract

**Background:**

Sulfamethoxazole (SMX, sulfonamide antibiotic) biodegradation by activated sludge communities (ASC) is still only partly understood. The present work is focusing on nine different bacteria species capable of SMX biodegradation that were isolated from SMX-acclimated ASC.

**Results:**

Initially 110 pure cultures, isolated from activated sludge, were screened by UV-absorbance measurements (UV-AM) for their SMX biodegradation potential. Identification via almost complete 16S rRNA gene sequencing revealed five *Pseudomonas* spp.*,* one *Brevundimonas* sp., one *Variovorax* sp. and two *Microbacterium* spp.. Thus seven species belonged to the phylum *Proteobacteria* and two to *Actinobacteria*. These cultures were subsequently incubated in media containing 10 mg L^-1^ SMX and different concentrations of carbon (sodium-acetate) and nitrogen (ammonium-nitrate). Different biodegradation patterns were revealed with respect to media composition and bacterial species. Biodegradation, validated by LC-UV measurements to verify UV-AM, occurred very fast with 2.5 mg L^-1^ d^-1^ SMX being biodegraded in all pure cultures in, for UV-AM modified, R2A-UV medium under aerobic conditions and room temperature. However, reduced and different biodegradation rates were observed for setups with SMX provided as co-substrate together with a carbon/nitrogen source at a ratio of DOC:N – 33:1 with rates ranging from 1.25 to 2.5 mg L^-1^ d^-1^.

**Conclusions:**

Media containing only SMX as carbon and nitrogen source proved the organisms’ ability to use SMX as sole nutrient source where biodegradation rates decreased to 1.0 – 1.7 mg L^-1^ d^-1^. The different taxonomically identified species showed specific biodegradation rates and behaviours at various nutrient conditions. Readily degradable energy sources seem to be crucial for efficient SMX biodegradation.

## Background

The widespread usage, disposal all around the world and a consumption of up to 200,000 t per year, makes the various groups of antibiotics an important issue for micropollutants risk assessment [1,2]. Their discharge and thus presence in the environment has become of major concern for environmental protection strategies. Antibiotics are designed to inhibit microorganisms and therefore influence microbial communities in different ecosystems [3,4]. Monitoring programs have already shown that antibiotics can be found nearly everywhere in the environment, even in concentrations up to μg L^-1^ leading to antibiotic resistance in organisms [5-9]. Antibiotic resistance genes might be transferred to human-pathogenic organisms by horizontal gene-transfer and become a serious issue, especially multidrug resistance in bacteria [10-12].

Sulfamethoxazole (SMX) is one of the most often applied antibiotics [13]. The frequent use of SMX results in wastewater concentrations up to μg L^-1^ and surface water concentrations in the ng L^-1^ scale [14-17]. Even in groundwater SMX was found at concentrations up to 410 ng L^-1^[16]. These SMX concentrations might be too low for inhibitory effects as the MIC_90_ for *M. tuberculosis* was found to be 9.5 mg L^-1^[18], but they might be high enough to function as signalling molecule to trigger other processes like quorum sensing in environmental microbial communities [19].

As shown by different studies [20-23], SMX can induce microbial resistances and reduce microbial activity and diversity arising the need for a better understanding of SMX biodegradation. SMX inflow concentrations in WWTPs in μg L^-1^ combined with often partly elimination ranging from 0% to 90% [4,6,15,24] result in high effluent discharge into the environment. To predict the extent of removal knowledge about the responsible biodegrading microorganisms is implicitly required to optimize environmental nutrient conditions for SMX removal and degradation rates. It is known that SMX can be removed by photodegradation occurring mainly in surface waters [25,26] and sorption processes in activated sludge systems [27]. However, biodegradation is, especially in WWTPs, probably the major removal process. Literature data focusing on SMX biodegradation in lab scale experiments with activated sludge communities and pure cultures showed a high fluctuation from almost complete SMX elimination (9, 28, 29) to hardly any removal of SMX (30). The determined SMX biodegradation potential was clearly affected by nutrient supply. Therefore this study’s emphasis is on clarifying the effect that addition of readily degradable carbon and/or nitrogen sources in some cases significantly enhanced SMX elimination (31) while in other cases supplementation showed no effect (28).

For this purpose pure culture were isolated from SMX-acclimated activated sludge communities and identified in respect to taxonomy and biodegradation capacity. Aerobic SMX biodegradation experiments with different species were carried out at various nutrient conditions to screen biodegradation potential and behaviour as a base for future research on biodegradation pathways.

## Results

### SMX biodegradation

#### Cultivation and evaluation of pure cultures biodegradation potential

Isolation of pure cultures was accomplished from SMX-acclimated ASC. Growth of cultures on solid R2A-UV media, spiked with 10 mg L^-1^ SMX, was controlled every 24 hours. All morphologically different colonies were streaked onto fresh R2A-UV agar plates, finally resulting in 110 pure cultures. For identification of potential SMX biodegrading cultures, all 110 isolates were inoculated in 20 mL MSM-CN media. SMX biodegradation was controlled every two days. After two days a decrease in absorbance was already detected in 5 cultures followed by 7 more at day 4 and 6 while the remaining cultures showed no change. The experiment was stopped after 21 days revealing no further SMX biodegrading culture. A 50% cutoff line defined a 50% decrease in UV-absorbance being significant enough to be sure that the corresponding organisms showed biodegradation. 12 organisms showed a decrease in absorbance greater than 50% of initial value and were defined as potential SMX biodegrading organisms. They were taxonomically identified and used for subsequent biodegradation experiments.

Additionally, biodegradation of these 12 identified isolates was validated by LC-UV (Table 1). For cost efficiency only initial and end concentrations of SMX in the media were determined as absorbance values did not change any more. A decrease in SMX concentration from initially 10 mg L^-1^ to below 5 mg L^-1^ was detected for all 12 isolates (Table 1) after 10 days of incubation. It was proven that only 3 cultures eliminated all 10 mg L^-1^ SMX completely while the residual SMX concentrations for the remaining cultures ranged from 0.23 to 4.35 mg L^-1^ after 10 days of incubation.

**Table 1:** Initial and end concentrations of SMX accomplished with 12 biodegrading pure culture isolates that were gained out of 110 cultures

**Pure culture**	**SMX conc. after 10 days [mg L**^**-1**^**]**
*Brevundimonas* sp. SMXB12	0.00
*Microbacterium* sp. SMXB24	0.00
*Microbacterium* sp. SMX348	0.00
*Pseudomonas* sp. SMX321	0.68
*Pseudomonas* sp. SMX330	0.68
*Pseudomonas* sp. SMX331	2.68
*Pseudomonas* sp. SMX 333*	1.09
*Pseudomonas* sp. SMX 336*	4.35
*Pseudomonas* sp. SMX 342*	1.09
*Pseudomonas* sp. SMX344*	0.23
*Pseudomonas* sp*.* SMX345	1.58
*Variovorax* sp*.* SMX332	3.53

*duplicate organisms. All but SMX344 were discarded.

Taxonomic identification succeeded with BLAST (http://blast.ncbi.nlm.nih.gov/Blast.cgi).

### Taxonomic and phylogenetic identification of pure cultures

**Figure 1:**
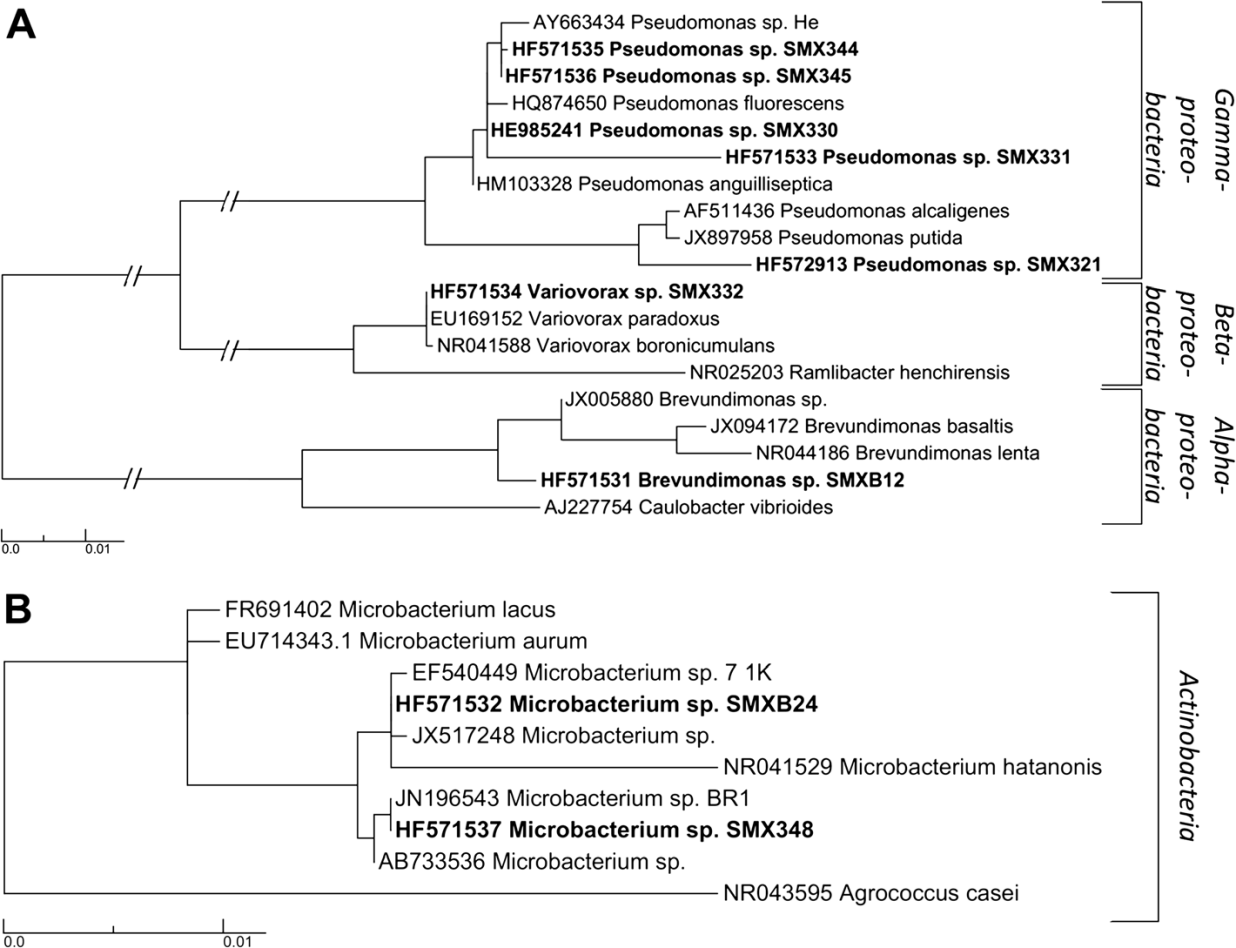
**Maximum likelihood-based trees reflecting the phylogeny and diversity of the isolated nine species capable of SMX biodegradation based on nearly complete 16S rRNA gene sequence comparisons.** Phylogenetic tree calculated for **A)***Pseudomonas* spp.*, Variovorax* spp. and *Brevundimonas* spp. and **B)** for *Microbacterium* spp.. The tree shows the sequences obtained in this study (bold text) and their next published relatives according to the NCBI database (plain text). Numbers preceding taxonomic names represent EMBL sequence accession numbers. Scale bar indicates 0.01% estimated sequence divergence.

All 12 cultures were identified by 16S rRNA gene sequence analysis to evaluate their phylogenetic position and closest relative. Four cultures, SMX 332, 333, 336 and 344, turned out to be the same organism closely related to *Pseudomonas* sp. He (AY663434) with a sequence similarity of 99%. Only SMX 344 was kept for further experiments as it showed fastest biodegradation in pre-tests (Table 1). Hence, a total of 9 different bacterial species with SMX biodegradation capacity were obtained. Their accession numbers, genus names and their closest relatives as found in the NCBI database (http://blast.ncbi.nlm.nih.gov/Blast.cgi), are shown as a maximum likelihood-based phylogenetic tree (Figure 1) evaluated with 16S rRNA gene sequence comparisons to calculate the most exact branching [28].

Seven of the nine isolates are affiliated within the phylum *Proteobacteria* represented by the classes *Alpha*-, *Beta*- and *Gammaproteobacteria*, while two belonged to the Phylum *Actinobacteria*.

The phylogenetic positions of the seven isolated pure cultures, affiliated within the phylum Proteobacteria, were located in the same tree (Figure 1A). Five different *Pseudomonas* spp. were identified and form two different clades representing a highly diverse group. *Pseudomonas* sp. SMX344 and 345 is building an individual cluster but belonged to the same group as SMX330 and 331. All four are closely related to *P. fluorescens* but SMX331 showed a remarkable difference. In contrast to the described *Pseudomonas* spp. above, *Pseudomonas* sp. SMX321 clusters together with *P. putida* and *P. alcaligenes* but forms an individual branch.

The other two *Proteobacteria* identified pure cultures belonged to the genera *Variovorax* (SMX332) and *Brevundimonas* (SMXB12). The isolated *Variovorax* SMX332 fell into the *Variovorax paradoxus/boronicumulans* group with a sequence similarity >99% to *V. paradoxus* (EU169152).

The *Brevundimonas* sp*.* SMXB12 was clearly separated from its closest relatives *Brevundimonas basaltis* and *B. lenta* and formed its own branch.

Both *Actinobacteria* affiliated pure cultures were identified as *Microbacterium* spp. and were embedded in a new phylogenetic tree as their phylogenetic position was too far from the other isolates (Figure 1B). The two isolated species were affiliated to two different clades clearly separated from *M. lacus* and *M. aurum. Microbacterium* sp*.* SMXB24 fell into the same group as *Microbacterium* sp*.* 7 1 K and *M. hatatonis* but the branch length clearly showed separation. *Microbacterium* sp*.* SMX348 was closely related with a sequence similarity of >99% to *Microbacterium* sp*.* BR1 which was found to biodegrade SMX in an acclimated membrane bioreactor [29].

### SMX biodegradation studies with pure cultures

Setups with sterile biomass (heat-killed) and without biomass (abiotic control) proved SMX to be stable under the operating conditions. Therefore sorption onto biomass or other materials was shown to be negligible. Photodegradation was excluded by performing all experiments in the dark.

**Figure 2:**
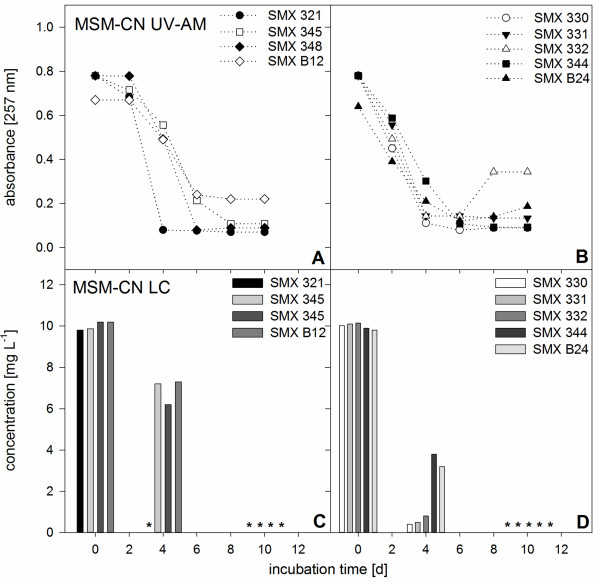
**Aerobic SMX biodegradation patterns of pure cultures in MSM-CN media. A, B)** measured with UV-AM**,** initial SMX concentration 10 mg L^-1^. **C, D)** LC-UV analyses of SMX concentrations in the used pure cultures in MSM-CN. Determination was performed at experimental startup, after 4 and 10 days to verify UV-AM values. Asterisks indicate measured values below limit of detection. Shown are mean values of SMX absorbance in duplicate experiments. Standard deviations were too low to be shown (<1%).

**Figure 3:**
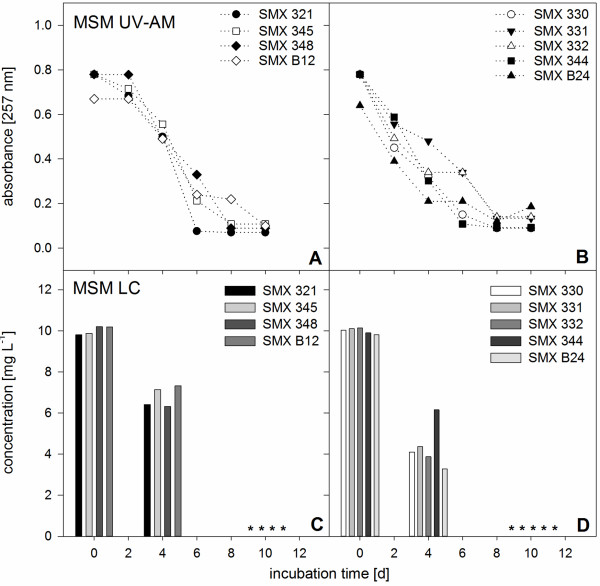
**Aerobic SMX biodegradation patterns of pure cultures in MSM media. A, B)** measured with UV-AM, initial SMX concentration 10 mg L^-1^. **C, D)** LC-UV analyses of SMX concentrations in the pure cultures in MSM at experimental startup, after 4 and 10 days to validate UV-AM. Asterisks indicate measured values below limit of detection. Shown are mean values of SMX absorbance in duplicate experiments. Standard deviations were too low to be shown (<1%).

To characterize biodegradation ability and rate and evaluate an optimal nutrient environment for SMX utilization of the isolated and identified 9 pure cultures, subsequent experiments were performed. In the presence of readily degradable carbon and/or nitrogen sources (Figures 2 and 3) SMX was faster biodegraded compared to setups with SMX as sole carbon/nitrogen source (Figure 3). 54 setups (three media for each of the 9 cultures in duplicate setups) with different nutrient compositions were set up and SMX biodegradation rates were evaluated using UV-AM values (Table 2). Different SMX biodegradation patterns were observed proving that the presence or absence of readily degradable and complex nutrients significantly influenced biodegradation.

**Table 2:** Biodegradation rates of the cultures able to biodegrade SMX

**Accession/isolate**	**Phylum**	Biodegradation rate* [mg L^**-1 **^**d**^**-1**^**]**		
		**R2A-UV**	**MSM-CN**	**MSM**
HF571531, *Brevundimonas* sp. SMXB12	*Proteobacteria*	2.5	1.7	1.0
HF571532, *Microbacterium* sp. SMXB24	*Actinobacteria*	2.5	1.25	1.25
HF571537, *Microbacterium* sp. SMX348	*Actinobacteria*	2.5	1.7	1.25
HF572913, *Pseudomonas* sp. SMX321	*Proteobacteria*	2.5	2.5	1.7
HE985241, *Pseudomonas* sp. SMX330	*Proteobacteria*	2.5	1.7	1.25
HF571533, *Pseudomonas* sp. SMX331	*Proteobacteria*	2.5	1.7	1.25
HF571535, *Pseudomonas* sp. SMX344	*Proteobacteria*	2.5	1.7	1.25
HF571536, *Pseudomonas* sp. SMX345	*Proteobacteria*	2.5	1.25	1.25
HF571534, *Variovorax* sp. SMX332	*Proteobacteria*	2.5	1.7	1.25

*calculated from duplicate experiments (n = 2). Standard deviations between duplicate setups were below 1% and are not shown.

Isolation was performed from an SMX-acclimated AS community, followed by identification with 16S rRNA sequencing. ENA accession numbers and species names are provided.

**Figure 4:**
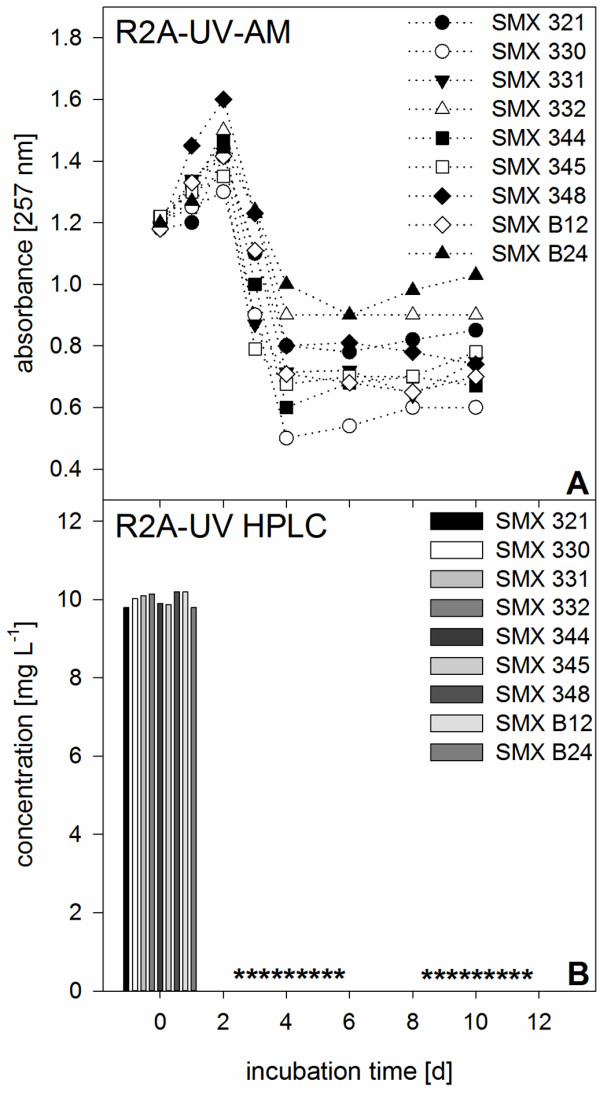
**Aerobic SMX biodegradation patterns of pure cultures in R2A-UV media. A)** measured with UV-AM, initial SMX concentration 10 mg L^-1^. **B)** LC-UV analyses of SMX concentrations within the nine pure cultures in R2A-UV media performed at experimental startup, after 4 and 10 days to verify the results of UV-AM. Asterisks indicate measured values below limit of detection. Shown are mean SMX absorbance values of duplicate experiments. Standard deviations were too low to be shown (<1%).

R2A-UV media were sampled once a day as it was assumed that biodegradation might be faster compared to the other two nutrient-poor media. Biodegradation rates of 2.5 mg L^-1^ d^-1^ were found for all nine species not showing any different biodegradation behaviors or patterns (Figure 4A). Although biomass growth affected background absorbance that increased with cell density, UV-AM could still be applied to monitor biodegradation as background absorbance was still in a measurable range.

In MSM-CN (Figure 2), offering only specific C- and N-sources, the biodegradation rates ranged from 1.25 to 2.5 mg L^-1^ d^-1^ (deviations between the duplicate setups were below 1%) showing clear differences for the different species, even for the five *Pseudomonas* spp.. While *Pseudomonas* sp. SMX321 biodegraded SMX with 2.5 mg L^-1^ d^-1^, *Pseudomonas* sp. SMX344 just showed a rate of 1.25 mg L^-1^ d^-1^. The same effect was found for the two *Microbacterium* spp.*.* While *Microbacterium* sp. SMXB12 removed SMX with 1.7 mg L^-1^ d^-1^, *Microbacterium* sp. SMX348 showed a removal of 1.25 mg L^-1^ d^-1^ only. Biodegradation pattern in MSM-CN of four isolates (SMX321, 345, 348 and B12) revealed a short lag phase of two days with no SMX removal (Figure 2A) while the other five were able to biodegrade SMX already after two days and showed a constant SMX removal during cultivation (Figure 2B).

In MSM (Figure 3), with SMX as sole C- and N-source, the removal rate of SMX was even lower. Biodegradation rates of 1.0 mg L^-1^ d^-1^ were found for *Brevundimonas* sp. SMXB12 while *Pseudomonas* sp. SMX321 showed 1.7 mg L^-1^ d^-1^. All other species showed removal rates of 1.25 mg L^-1^ d^-1^. These experiments with SMX as sole C/N-source proved that it could serve as nutrient source but with up to 2.5-fold reduced biodegradation rates. Biodegradation pattern in MSM was similar to that in MSM-CN with a lag phase of two days for the four isolates SMX321, 345, 348 and B12 (Figure 3A) and no lag phase for the isolates SMX 330, 331, 332, 344, and B24 starting to utilize SMX already after two days (Figure 3B). In general it was found that the five *Pseudomonas* spp. and the two *Microbacterium* spp*.* did not show the same biodegradation behavior. At least one member of each group always showed a lag phase while the other immediately started SMX biodegradation.

As UV-AM revealed sufficient to monitor SMX biodegradation (Table 1) LC-UV measurements were only performed at the start of the experiment, day 4 and at day 10 as control measurement (Figures 3B, 4C, D). LC-UV showed that in R2A-UV all cultures removed 10 mg L^-1^ SMX in 4 days (Figure 2B) while in MSM-CN only *Pseudomonas* sp. SMX321 removed all SMX within 4 days (Figure 3C). The remaining 8 cultures still showed residual SMX concentrations from 0.4 to 7.3 mg L^-1^ and complete SMX elimination was achieved only at day 10 (Figure 3C, D). In MSM after 4 days SMX was still present in all nine cultures in concentrations above 3.6 mg L^-1^ and only after 10 days SMX was below the limit of detection (Figure 4C, D). LC-UV values could be compared to UV-AM values and proved this simple approach to be applicable for screening SMX biodegradation.

## Discussion and conclusions

This study focused on the cultivation of pure culture SMX biodegrading organisms to perform specific biodegradation experiments. It is known that cultivation, especially on solid media, is affected with the problem described as “viable but non cultivable” (VBNC) [30,31]. Solid media being implicitly required for the isolation of pure cultures is for sure limited in its cultivation efficiency mainly due to reduced water content and different or inappropriate nutrient conditions. Thus only a low percentage of around 1% of the active organisms in environmental samples [32] and around 15% from activated sludge can be cultivated [33,34]. In this study 9 different isolates out of 110 pure cultures were obtained that showed SMX biodegradation. This quite high percentage of almost 10% was only possible with a two-step SMX-acclimation experiment that was conducted to increase the chance to cultivate SMX biodegrading organisms by applying a strong selective pressure using 10 mg L^-1^ SMX in the media. Furthermore, R2A medium that is known to work well for isolation of aquatic organisms [35] was applied for the cultivation of bacteria being assumed to be at least SMX-resistant when growth was observed on SMX-reinforced R2A. However, a lot more organisms compared to those cultivated in this study might be present in activated sludge capable of SMX biodegradation. These VBNCs might be taxonomically characterized by culture-independent methods, e.g. restriction fragment length polymorphism screening [36,37]. However, for our focus on linking biodegradation patterns, rates and nutrient utilization to specific species these methods were not feasible. Only with actively biodegrading pure cultures a clear and precise coherence between SMX biodegradation and taxonomically identified species is possible. As a final goal, pure cultures would allow to analyze species-specific biodegradation products and thus determine potential SMX biodegradation pathways. Applying that knowledge to WWTP techniques would provide a strategy to selectively enhance biodegrading species in activated sludge systems improving and stabilizing SMX removal efficiency.

Therefore phylogenetic identification of potential SMX biodegrading species is implicitly required. As shown in this study five of the nine SMX biodegrading species found belonged to the genus *Pseudomonas* confirming this group to play an important role for the biodegradation of micropollutants. This was proved for e.g. acetaminophen or chlorinated compounds by many other studies [38-40]. Additionally, two isolates SMXB24 and SMX348 were identified as *Microbacterium* sp.. It was shown that *Microbacterium* sp. SMXB24 is closely related to *Microbacterium* sp. 7 1 K, an organism that was found to be related with phytoremediation. The second *Microbacterium* sp. SMX348 is closely related to *Microbacterium* sp. BR1 which was isolated from an acclimated SMX biodegrading membrane bioreactor, proving this species’ crucial role for the biodegradation of SMX [29]. In addition the general potential of different *Microbacteria* species for the biodegradation of xenobiotic compounds has been highlighted in the literature [41,42]. Also *Variovorax paradoxus*, closely related to the isolated *Variovorax* sp. SMX332, is known from literature to be capable of biodegrading a large variety of pollutants including sulfolene and other heterocyclic compounds [43]. Therefore it seems likely that the isolated *Variovorax* sp. SMX332 might also be able to biodegrade SMX. Finally, also for the group *Brevundimonas* spp. some literature data exist proving that these organisms might play a role in the removal of antibiotics [44].

Taxonomic identification was followed by observing influences on biodegradation rate and efficiency due to the availability of nutrients. Biodegradation rates decreased with reduced nutrient content from the complex R2A-UV over nutrient-poor MSM-CN and MSM media and more time was needed to remove SMX. MSM media contained SMX as sole carbon and nitrogen source at a concentration of 10 mg L^-1^ and thus provided just around 4.8 mg L^-1^ carbon and 1.7 mg L^-1^ nitrogen. These conditions, with SMX being the only nutrient in MSM, showed an effect on biodegradation and reduced removal efficiency but proved the organisms’ ability to utilize SMX as sole nutrient and/or energy source. However, this indicates that complex nutrients and higher nutrient concentrations seem to have a positive effect on biodegradation due to co-metabolic [45] or diauxic effects [46] as the very high SMX removal rates of 2.5 mg L^-1^ d^-1^ confirmed that they were significantly higher than the one of 0.0079 mg L^-1^ d^-1^ found in a previous study [47].

In general, SMX biodegradation might be based more on a diauxic process, i.e. readily degradable nutrients are used up first followed by SMX utilization, rather than real co-metabolism, i.e. two substrates are used up in parallel when provided together, as experiments with R2A-UV media showed. A strong increase in UV-AM, attributed to biomass growth due to a fast nutrient consumption provided by the complex R2A-UV media, was followed by a rapid SMX elimination. In MSM-CN or MSM, as the nutrients concentrations were too low to foster excessive biomass growth, such an increase was not observed . Even at low cell densities SMX was rapidly removed proving that biomass concentration is not as important as cellular activity. Therefore, the higher removal rates in presence of sufficient nutrients also showed that SMX biodegradation was a rapid and complex metabolic process.

Therefore, information about the biodegradation potential of the isolated bacterial strains with respect to the availability of nutrients might increase the elimination efficiency in WWTPs as the treatment process could be specifically adapted to the needs of the biodegrading species.

For future research, the availability of isolated species will allow screening for biodegradation intermediates and/or stable metabolites and determination of species-specific biodegradation pathways. To date only few data on SMX metabolites such as 3-amino-5-methyl-isoxazole found in SMX degrading activated sludge communities [48] and hydroxy-N-(5-methyl-1,2-oxazol-3-yl)benzene-1-sulfonamide detected in an SMX degrading consortium of fungi and *Rhodococcus rhodochrous* exists [45]. Further research is also needed to screen for the nutrient influence on metabolite formation, i.e. if the isolated pure cultures produce different metabolites due to changing nutrient conditions.

## Methods

### Chemicals and glassware

Sulfamethoxazole (SMX, 99.8% purity) was purchased from Sigma Aldrich (Steinheim, Germany), all other organic media components were from Merck KGaA (Darmstadt, Germany) while the inorganic media components were purchased from VWR (Darmstadt, Germany). High-purity water was prepared by a Milli-Q system (Millipore, Billerica, MA, USA). All glassware used was procured from Schott AG (Mainz, Germany) and pre-cleaned by an alkaline detergent (neodisher®, VWR Darmstadt, Germany) followed by autoclaving for 20 min at 121°C.

### Activated sludge sampling

Activated sludge (AS) was taken as grab sample from stage 1 of a 2-stage municipal conventional activated sludge plant (CAS-M), located near the city of Munich, Germany and treating 1 million populations equivalents. Stage 1 is the high load stage with a food to microorganism ratio of 0.64 kg BOD_5_ kg^-1^ MLSS^-1^. The influent consists of municipal and industrial wastewater (1:1). 500 mL AS (SMX concentration 600 ng L^-1^) were collected in pre-cleaned 1 L glass bottles, stored at 4°C and used within 24 h for inoculation of the different setups.

### Experimental setup

#### SMX acclimated ASC

Evaluation of AS biodegradation potential obtained from the WWTP, was performed in 150 mL R2A-UV media (casein peptone 1,000 mg L^-1^, glucose 500 mg L^-1^, potassium phosphate 300 mg L^-1^, soluble starch 300 mg L^-1^, DOC:N ratio 7:1, pH 7.4), spiked with 10 mg L^-1^ SMX to apply a high selective pressure. Non-SMX-resistant organisms were ruled out and the chance to obtain SMX biodegrading organisms was increased in subsequent isolation steps. After biodegradation occurred the experiment was stopped and the remaining biomass was used to inoculate a second setup under the same conditions to further decrease microbial diversity and favor SMX-resistant/biodegrading organisms. After the second setup showed biodegradation, the experiment was stopped and the biomass used for cultivation of SMX biodegrading organisms on solid R2A-UV media (1.5% agar supply). SMX removal was determined by UV-absorbance measurements (UV-AM) as fast pre-screening method for biodegradation (see 2.4.1).

#### Cultivation and isolation of pure cultures

Pure cultures were successfully cultivated and isolated from SMX-acclimated biodegrading ASC. 200 μL AS was plated on solid R2A-UV media containing 10 mg L^-1^ SMX to inhibit growth of non-resistant bacteria and foster growth of potential SMX-resistant/biodegrading organisms. After cultures were observed on solid media they were isolated and further purified by streaking on new plates resulting in 110 isolates. These were used for inoculation of 100 mL setups with 20 mL MSM-CN media (KH_2_PO_4_ 80 mg L^-1^, K_2_HPO_4_ 200 mg L^-1^, Na_2_HPO_4_ 300 mg L^-1^, MgSO_4_*7 H_2_O 20 mg L^-1^, CaCl*2 H_2_O 40 mg L^-1^, FeCl_3_*6 H_2_O 0.3 mg L^-1^, sodium acetate 300 mg L^-1^ and NH_4_NO_3_ 7.5 mg L^-1^, DOC:N ratio 33:1, pH 7.4) spiked with 10 mg L^-1^ SMX. Setups were monitored with UV-AM (see 2.4.1) for possible biodegradation. Isolates showing biodegradation were further identified by 16S rRNA gene sequence analysis (see 2.5).

#### Biodegradation setups with pure cultures

Batch experiments were performed to A) screen for biodegradation potential in the isolated cultures and B) determine differences in SMX biodegradation pattern and rate concerning the availability of nutrients. Three media, R2A-UV, MSM-CN and MSM (as MSM-CN but without sodium acetate and NH_4_NO_3_) were used and inoculated with pure cultures in 100 mL setups filled with 20 mL of media spiked with 10 mg L^-1^ SMX. Duplicate setups (n = 2) including sterile, i.e. autoclaved biomass and abiotic, i.e. without biomass, controls for each medium were prepared. Aerobic conditions and photolysis prevention were ensured by shaking at 150 rpm on an orbital shaker in the dark.

The setups were sampled once a day for MSM-CN and MSM media and twice a day for R2A-UV, by taking 1 mL supernatant after half an hour of sedimentation that was sufficient to ensure not to withdraw much biomass. 200 μL was used for UV-AM and 800 μL for LC-UV measurements.

### Analyses of sulfamethoxazole

#### UV-AM

200 μL were taken from the setups and directly used for UV-AM as described elsewhere (Herzog et al., submitted) with the following changes applied. Calibration was performed with 1.0, 5.0, 10.0 and 15.0 mg L^-1^ SMX in high-purity water and the used media to evaluate measurement reliability and background absorbance. 96 well UV-star plates from Greiner Bio-One (Greiner Bio-One GmbH, Frickenhausen, Germany) filled with 200 μL were used for measurements and analyzed with an automated plate reader (EnSpire® Multimode Plate Reader, Perkin Elmer, Rodgau, Germany). Each measurement included an SMX blank (media with SMX but without organisms) was measured to detect changes over time as well as a blank (media without SMX) to detect background absorbance.

#### LC-UV analysis

800 μL samples obtained from the setups were centrifuged (10 min, 8000 g, 20°C), filtrated through a 0.45 μm membrane filter to remove cellular debris and biomass and filled into sterile glass flasks. Flasks were stored at-20°C before analysis.

Analysis was performed with a Dionex 3000 series HPLC system (Dionex, Idstein, Germany), equipped with an auto sampler. A DAD scanning from 200 to 600 nm was applied to detect and quantify SMX. Chromatographic separation was achieved on a Nucleosil 120-3 C18 column (250 mm × 3.0 mm i.d., 3 μm particle size) from Macherey Nagel (Düren, Germany) at a column temperature of 25°C. The applied mobile phases were acetonitrile (AN) and water (pH 2.5 using phosphoric acid). The gradient used for the first 5 min was 7% AN followed by 7-30% AN from 5-18 min, 30% AN for minutes 18-30 and finally 7% AN for minutes 30-35. The solvent flow rate was 0.6 mL min^-1^. The column was allowed to equilibrate for 5 min between injections. Limit of quantification and limit of detection were 0.1 mg L^-1^ and 0.03 mg L^-1^, respectively.

### Taxonomic and phylogenetic identification of isolated pure cultures by 16S rRNA gene sequence analysis

DNA of SMX biodegrading organisms was extracted by a standard phenol/chloroform/CTAB extraction method. 16S rRNA gene was subsequently amplified via standard PCR using universal bacterial primers 27f (5-AGA GTT TGA TCM TGG CTC AG-3) and 1492r (5-TAC GGY TAC CTT GTT ACG ACT T-3) [49]. All cultures were sent to MWG Operon (Ebersberg, Germany) for sequencing using again primers 27f and 1492r and resulting in nearly full length 16S rRNA gene sequences. Sequences were analyzed with and submitted to European Nucleotide Archive (http://www.ebi.ac.uk/ena/) to receive accession numbers (Table 2).

Subsequent phylogenetic analysis was accomplished with the sequences using the alignment and tree calculation methods of the ARB software package [50]. The nearly complete 16S rRNA gene sequences of the species isolated in this study and their corresponding published closest relatives (http://blast.ncbi.nlm.nih.gov/Blast.cgi) were added to an existing ARB-alignment for the 16S rRNA gene sequence. Alignment was performed with the CLUSTAL W implemented in ARB. Phylogenetic trees of the 16S rRNA gene sequences were calculated based on maximum likelihood.

## Competing interest

The authors declare that there are no competing interests.

## Authors’ contributions

BH drafted the manuscript, designed and carried out the biodegradation experiments. HL reviewed the manuscript. HH and EM conceived of the study, participated in its coordination and helped to review the manuscript. All authors read and approved the final manuscript.

## References

[B1] Kümmerer K Pharmaceuticals in the environment: sources, fate, effects, and risks 2004 2 Berlin, Heidelberg, Germany: Springer

[B2] Kümmerer K Pharmaceuticals in the environment. 3rd, Revised and enlarged Edition edn 2008 Berlin, Heidelberg, Germany: Springer

[B3] Baran W Sochacka J Wardas W Toxicity and biodegradability of sulfonamides and products of their photocatalytic degradation in aqueous solutions Chemosphere 2006 65 1295 1299 10.1016/j.chemosphere.2006.04.040 16750553

[B4] Xu B Mao D Luo Y Xu L Sulfamethoxazole biodegradation and biotransformation in the water-sediment system of a natural river Bioresour Technol 2011 102 7069 7076 10.1016/j.biortech.2011.04.086 21596556

[B5] Heberer T Occurrence, fate, and removal of pharmaceutical residues in the aquatic environment: a review of recent research data Toxicol Lett 2002 131 5 17 10.1016/S0378-4274(02)00041-3 11988354

[B6] Ternes T Joss A Human pharmaceuticals, hormones and fragrances the challenge of micropollutants in urban water management 2007

[B7] Kümmerer K Antibiotics in the aquatic environment-a review-part I Chemosphere 2009 75 417 434 10.1016/j.chemosphere.2008.11.086 19185900

[B8] Kümmerer K Antibiotics in the aquatic environment-a review-part II Chemosphere 2009 75 435 441 10.1016/j.chemosphere.2008.12.006 19178931

[B9] Pérez S Eichhorn P Aga DS Evaluating the biodegradability of sulfamethazine, sulfamethoxazole, sulfathiazole, and trimethoprim at different stages of sewage treatment Environ Toxicol Chem 2005 24 1361 1367 10.1897/04-211R.1 16117111

[B10] Hoa PTP Managaki S Nakada N Takada H Shimizu A Anh DH Viet PH Suzuki S Antibiotic contamination and occurrence of antibiotic-resistant bacteria in aquatic environments of northern Vietnam Sci Total Environ 2011 409 2894 2901 10.1016/j.scitotenv.2011.04.030 21669325

[B11] Agerso Y Petersen A The tetracycline resistance determinant Tet 39 and the sulphonamide resistance gene sulII are common among resistant Acinetobacter spp. isolated from integrated fish farms in Thailand J Antimicrob Chemother 2007 59 23 27 17095527 10.1093/jac/dkl419

[B12] Szczepanowski R Linke B Krahn I Gartemann K-H Gützkow T Eichler W Pühler A Schlüter A Detection of 140 clinically relevant antibiotic-resistance genes in the plasmid metagenome of wastewater treatment plant bacteria showing reduced susceptibility to selected antibiotics Microbiology 2009 155 2306 2319 10.1099/mic.0.028233-0 19389756

[B13] Cavallucci S Top 200: What’s topping the charts in prescription drugs this year Pharmacy practice, Canadian Healthcare Network 2007

[B14] Benotti MJ Trenholm RA Vanderford BJ Holady JC Stanford BD Snyder SA Pharmaceuticals and endocrine disrupting compounds in US drinking water Environ Sci Technol 2008 43 597 603 19244989 10.1021/es801845a

[B15] Miège C Choubert J Ribeiro L Eusèbe M Coquery M Fate of pharmaceuticals and personal care products in wastewater treatment plants-Conception of a database and first results Environ Pollut 2009 157 1721 1726 10.1016/j.envpol.2008.11.045 19201071

[B16] Sacher F Lange FT Brauch HJ Blankenhorn I Pharmaceuticals in groundwaters: analytical methods and results of a monitoring program in Baden-Wurttemberg, Germany J Chromatogr 2001 938 199 210 10.1016/S0021-9673(01)01266-3 11771839

[B17] Onesios K Yu J Bouwer E Biodegradation and removal of pharmaceuticals and personal care products in treatment systems: a review Biodegradation 2009 20 441 466 10.1007/s10532-008-9237-8 19112598

[B18] Huang T-S Kunin CM Yan B-S Chen Y-S Lee SS-J Syu W Susceptibility of Mycobacterium tuberculosis to sulfamethoxazole, trimethoprim and their combination over a 12 year period in Taiwan J Antimicrob Chemother 2012 67 633 637 10.1093/jac/dkr501 22127584

[B19] Fajardo A Martínez JL Antibiotics as signals that trigger specific bacterial responses Curr Opin Microbiol 2008 11 161 167 10.1016/j.mib.2008.02.006 18373943

[B20] Jiang X Shi L Distribution of tetracycline and trimethoprim/sulfamethoxazole resistance genes in aerobic bacteria isolated from cooked meat products in Guangzhou, China Food Control 2013 30 30 34 10.1016/j.foodcont.2012.06.042

[B21] Liu F Wu J Ying G-G Luo Z Feng H Changes in functional diversity of soil microbial community with addition of antibiotics sulfamethoxazole and chlortetracycline Appl Microbiol Biotechnol 2012 95 1615 1623 10.1007/s00253-011-3831-0 22205443

[B22] Gutiérrez I Watanabe N Harter T Glaser B Radke M Effect of sulfonamide antibiotics on microbial diversity and activity in a Californian Mollic Haploxeralf J Soils Sed 2010 10 537 544 10.1007/s11368-009-0168-8

[B23] Collado N Buttiglieri G Marti E Ferrando-Climent L Rodriguez-Mozaz S Barceló D Comas J Rodriguez-Roda I Effects on activated sludge bacterial community exposed to sulfamethoxazole Chemosphere 2013 93 99 106 10.1016/j.chemosphere.2013.04.094 23726012

[B24] Göbel A McArdell CS Joss A Siegrist H Giger W Fate of sulfonamides, macrolides, and trimethoprim in different wastewater treatment technologies Sci Total Environ 2007 372 361 371 10.1016/j.scitotenv.2006.07.039 17126383

[B25] Niu J Zhang L Li Y Zhao J Lv S Xiao K Effects of environmental factors on sulfamethoxazole photodegradation under simulated sunlight irradiation: kinetics and mechanism J Environ Sci 2013 25 1098 1106 10.1016/S1001-0742(12)60167-3 24191598

[B26] Trovó AG Nogueira RFP Agüera A Sirtori C Fernández-Alba AR Photodegradation of sulfamethoxazole in various aqueous media: persistence, toxicity and photoproducts assessment Chemosphere 2009 77 1292 1298 10.1016/j.chemosphere.2009.09.065 19879626

[B27] Hyland KC Dickenson ERV Drewes JE Higgins CP Sorption of ionized and neutral emerging trace organic compounds onto activated sludge from different wastewater treatment configurations Water Res 2012 46 1958 1968 10.1016/j.watres.2012.01.012 22316557

[B28] Ludwig W Klenk H-P Boone D, Castenholz R Overview: a phylogenetic backbone and taxonomic framework for procaryotic systematics Bergey’s manual® of systematic bacteriology 2001 New York: Springer 49 65

[B29] Bouju H Ricken B Beffa T Corvini PF Kolvenbach BA Isolation of bacterial strains capable of sulfamethoxazole mineralization from an acclimated membrane bioreactor Appl Environ Microbiol 2012 78 277 279 10.1128/AEM.05888-11 22020509 PMC3255614

[B30] Jiang Q Fu B Chen Y Wang Y Liu H Quantification of viable but nonculturable bacterial pathogens in anaerobic digested sludge Appl Microbiol Biotechnol 2013 97 6043 6050 10.1007/s00253-012-4408-2 22996281

[B31] Wagner M Assmus B Hartmann A Hutzler P Amann R In situ analysis of microbial consortia in activated sludge using fluorescently labelled, rRNA-targeted oligonucleotide probes and confocal scanning laser microscopy J Microsc 1994 176 181 187 10.1111/j.1365-2818.1994.tb03513.x 7532718

[B32] Vartoukian SR Palmer RM Wade WG Strategies for culture of ‘unculturable’ bacteria FEMS Microbiol Lett 2010 309 1 7 20487025 10.1111/j.1574-6968.2010.02000.x

[B33] Wagner M Amann R Lemmer H Schleifer KH Probing activated sludge with oligonucleotides specific for proteobacteria: inadequacy of culture-dependent methods for describing microbial community structure Appl Environ Microbiol 1993 59 1520 1525 8517747 10.1128/aem.59.5.1520-1525.1993 PMC182113

[B34] Snaidr J Amann R Huber I Ludwig W Schleifer KH Phylogenetic analysis and in situ identification of bacteria in activated sludge Appl Environ Microbiol 1997 63 2884 2896 9212435 10.1128/aem.63.7.2884-2896.1997 PMC168584

[B35] Reasoner DJ Geldreich EE A new medium for the enumeration and subculture of bacteria from potable water Appl Environ Microbiol 1985 49 1 7 3883894 10.1128/aem.49.1.1-7.1985 PMC238333

[B36] Chiellini C Munz G Petroni G Lubello C Mori G Verni F Vannini C Characterization and comparison of bacterial communities selected in conventional activated sludge and membrane bioreactor pilot plants: a focus on nitrospira and planctomycetes bacterial phyla Curr Microbiol 2013 67 77 90 10.1007/s00284-013-0333-6 23420462

[B37] Wells GF Park H-D Eggleston B Francis CA Criddle CS Fine-scale bacterial community dynamics and the taxa-time relationship within a full-scale activated sludge bioreactor Water Res 2011 45 5476 5488 10.1016/j.watres.2011.08.006 21875739

[B38] Larcher S Yargeau V Biodegradation of sulfamethoxazole by individual and mixed bacteria Appl Microbiol Biotechnol 2011 91 211 218 10.1007/s00253-011-3257-8 21499763

[B39] De Gusseme B Vanhaecke L Verstraete W Boon N Degradation of acetaminophen by delftia tsuruhatensis and pseudomonas aeruginosa in a membrane bioreactor Water Res 2011 45 1829 1837 10.1016/j.watres.2010.11.040 21167545

[B40] Tezel U Tandukar M Martinez RJ Sobecky PA Pavlostathis SG Aerobic biotransformation of n-tetradecylbenzyldimethylammonium chloride by an enriched pseudomonas spp. Community Environ Sci Technol 2012 46 8714 8722 10.1021/es300518c 22794799

[B41] Shiomi N Ako M Biodegradation of melamine and cyanuric acid by a newly-isolated microbacterium strain Adv Microbiol 2012 2 303 309 10.4236/aim.2012.23036

[B42] Chunming W Chunlian LIDW Biodegradation of naphthalene, phenanthrene, anthracene and pyrene by microbacterium sp. 3-28 Chin J Appl Environ Biol 2009 3 017

[B43] Satola B Wübbeler J Steinbüchel A Metabolic characteristics of the species variovorax paradoxus Appl Microbiol Biotechnol 2013 97 541 560 10.1007/s00253-012-4585-z 23192768

[B44] Islas-Espinoza M Reid B Wexler M Bond P Soil bacterial consortia and previous exposure enhance the biodegradation of sulfonamides from Pig manure Microb Ecol 2012 64 140 151 10.1007/s00248-012-0010-5 22286498

[B45] Gauthier H Yargeau V Cooper DG Biodegradation of pharmaceuticals by rhodococcus rhodochrous and aspergillus niger by co-metabolism Sci Total Environ 2010 408 1701 1706 10.1016/j.scitotenv.2009.12.012 20089297

[B46] Cohen GN Bacterial growth Microbial biochemistry 2011 Dordrech, Netherlands: Springer 1 10

[B47] Yang S-F Lin C-F Wu C-J Ng K-K Yu-Chen Lin A Andy Hong P-K Fate of sulfonamide antibiotics in contact with activated sludge-sorption and biodegradation Water Res 2012 46 1301 1308 10.1016/j.watres.2011.12.035 22227239

[B48] Müller E Schüssler W Horn H Lemmer H Aerobic biodegradation of the sulfonamide antibiotic sulfamethoxazole by activated sludge applied as co-substrate and sole carbon and nitrogen source Chemosphere 2013 92 969 978 10.1016/j.chemosphere.2013.02.070 23611245

[B49] Weisburg WG Barns SM Pelletier DA Lane DJ 16S ribosomal DNA amplification for phylogenetic study J Bacteriol 1991 173 697 703 1987160 10.1128/jb.173.2.697-703.1991 PMC207061

[B50] Ludwig W Strunk O Westram R Richter L Meier H Buchner A Lai T Steppi S Jobb G Yadhukumar ARB: a software environment for sequence data Nucleic Acids Res 2004 32 1363 1371 10.1093/nar/gkh293 14985472 PMC390282

